# Visual short-term memory binding deficit in familial Alzheimer's disease

**DOI:** 10.1016/j.cortex.2016.01.015

**Published:** 2016-05

**Authors:** Yuying Liang, Yoni Pertzov, Jennifer M. Nicholas, Susie M.D. Henley, Sebastian Crutch, Felix Woodward, Kelvin Leung, Nick C. Fox, Masud Husain

**Affiliations:** a Dementia Research Centre, Department of Neurodegenerative Diseases, UCL Institute of Neurology, London, UK; b Department of Psychology, The Hebrew University of Jerusalem, Israel; c Department of Medical Statistics, London School of Hygiene and Tropical Medicine, London, UK; d Nuffield Department of Clinical Neurosciences, University of Oxford, UK; e Department of Experimental Psychology, University of Oxford, UK

**Keywords:** Working memory, Visual short-term memory, Relational binding, Hippocampus, Medial temporal lobe, WM, working memory;, MTL, medial temporal lobe, HAD, hospital anxiety and depression scale, NART, National Adult Reading Test, TR, repetition time, TE, echo time, TI, inversion time, FOV, field of view

## Abstract

Long-term episodic memory deficits in Alzheimer's disease (AD) are well characterised but, until recently, short-term memory (STM) function has attracted far less attention. We employed a recently-developed, delayed reproduction task which requires participants to reproduce precisely the remembered location of items they had seen only seconds previously. This paradigm provides not only a continuous measure of localization error in memory, but also an index of *relational binding* by determining the frequency with which an object is misplaced to the location of one of the other items held in memory. Such *binding errors* in STM have previously been found on this task to be sensitive to medial temporal lobe (MTL) damage in focal lesion cases. Twenty individuals with pathological mutations in presenilin 1 or amyloid precursor protein genes for familial Alzheimer's disease (FAD) were tested together with 62 healthy controls. Participants were assessed using the delayed reproduction memory task, a standard neuropsychological battery and structural MRI.

Overall, FAD mutation carriers were worse than controls for object identity as well as in gross localization memory performance. Moreover, they showed greater misbinding of object identity and location than healthy controls. Thus they would often mislocalize a correctly-identified item to the location of one of the other items held in memory. Significantly, *asymptomatic* gene carriers – who performed similarly to healthy controls on standard neuropsychological tests – had a specific impairment in object-location binding, despite intact memory for object identity and location. Consistent with the hypothesis that the hippocampus is critically involved in relational binding regardless of memory duration, decreased hippocampal volume across FAD participants was significantly associated with deficits in object-location binding but not with recall precision for object identity or localization. Object-location binding may therefore provide a sensitive cognitive biomarker for MTL dysfunction in a range of diseases including AD.

## 1 Introduction

Memory impairment is a central, defining feature of Alzheimer's disease (e.g., Dubois et al., 2007, McKhann et al., 1984). While long-term, episodic memory dysfunction has been widely documented (e.g., Greene et al., 1996, Hodges, 2000), far less attention has been devoted to short-term memory (STM) deficits in the condition. STM is one component of working memory (WM), the cognitive system that underlies our ability to temporarily *maintain* as well as *manipulate* information when it is no longer accessible in the environment (Baddeley, 2010, Baddeley and Hitch, 1974, D'Esposito and Postle, 2015, Postle, 2006). The ability to hold onto information over short periods of time has a pivotal role in almost every cognitive task. Earlier investigations of Alzheimer's disease reported a general deficit in the central executive component of WM (Baddeley et al., 1986, Baddeley et al., 1991), rather than in maintenance. More recent work, however, has emphasised a reduction in WM capacity, highlighting a difficulty in storage (Stopford, Thompson, Neary, Richardson, & Snowden, 2012) linked to atrophy in temporo-parietal regions (Snowden et al., 2007, Stopford et al., 2012).

Is there any particular aspect of maintenance that is disrupted in Alzheimer's disease? One important line of research has provided evidence that the ability to bind object features together in WM might be critically affected. In their pioneering studies, Parra and colleagues reported that binding in visual short-term memory (VSTM) of simple object features such as colour and shape or colour and colour is selectively disrupted in Alzheimer's disease (Parra et al., 2009, Parra et al., 2010, Parra et al., 2011). These studies employed a version of the change detection paradigm, which can be used to measure VSTM *capacity* – the number of items an individual can remember over short durations. Change detection depends upon a binary response: either something is remembered correctly or it is not. But just because an individual fails to recall an item correctly does not necessarily mean that it was completely abolished from memory. More recently, an alternative theoretical and empirical approach to VSTM has been developed to investigate the *resolution* with which items are retained (for a review see Ma, Husain, & Bays, 2014).

Instead of asking participants to report whether they detect a change between sample and test arrays, they are requested to reproduce a feature of an object using a continuous, *analogue* response space (Bays et al., 2009, Gorgoraptis et al., 2011, Wilken and Ma, 2004). Such delayed reproduction tasks measure *precision* of recall and provide an index of the quality of memory representation. Delayed reproduction tasks have now been reported to be more sensitive than conventional span measures of WM which also index only the number of items held in memory (Zokaei, Burnett Heyes, Gorgoraptis, Budhdeo, & Husain, 2014).

Importantly, such WM precision tasks also provide a means to dissect out sources of error contributing to the pattern of performance (see Ma et al., 2014). Errors can potentially arise from several different factors. First, they may be due to variability in memory for the *probed* item – the quality with which it is stored. Second, errors may be random because, on some trials, participants simply guess, e.g., they might fail to encode an item because they were not paying attention. Finally, error can arise from misreporting features of *non-probed* items that were presented in the memory array, instead of reporting the features that belonged to the probed item. In other words, recall may be systematically corrupted by features of other objects retained in VSTM – a deficit in maintaining correctly the feature bindings of an item.

Pertzov et al. recently introduced a delayed reproduction paradigm that measures precision of recall for ‘what was where?’ They used it to investigate the nature of WM deficits in individuals with focal medial temporal lobe (MTL) damage due to voltage-gated potassium channel antibody (VGKC-Ab) mediated limbic encephalitis (Pertzov et al., 2013). These patients showed a specific impairment in binding object identity to location but had no difficulty remembering the identities and locations on their own. Thus when participants mislocalized objects, their reports were often clustered around the locations of other objects in the array rather than occurring randomly (Pertzov et al., 2012, Pertzov et al., 2013). As damage in VGKC-Ab limbic encephalitis involves the hippocampus both on pathological and neuroimaging grounds (Khan et al., 2009, Pertzov et al., 2013), it has been suggested that the hippocampus or adjacent MTL structures might be crucial for feature binding in WM (Pertzov et al., 2013).

This proposal would be consistent with several lines of evidence that the hippocampus plays a key role in *relational memory*, binding together relationships of distinct elements in episodic memory (Eichenbaum, 2006, Konkel et al., 2008, Mayes et al., 2007). More recent findings, from lesion and functional imaging studies, have suggested that it also plays a role in relational binding in STM, e.g., in binding object identity to location (Hannula, Tranel, & Cohen, 2006; Hannula et al., 2015, Libby et al., 2014, Olson et al., 2006, Watson et al., 2013).

In Alzheimer's disease, the hippocampus is one of the earliest structures affected by pathology (Bateman et al., 2012, Braak and Braak, 1991, Fox et al., 1996, Fox et al., 1996, Reiman et al., 2012). Indeed, longitudinal studies in familial Alzheimer's disease (FAD) cases have shown that progressive hippocampal atrophy can be detected many years before the diagnosis of dementia and in the asymptomatic stage of the disease (Fox et al., 1996, Fox et al., 1996, Ridha et al., 2006, Schott et al., 2003). Interestingly, however, the types of VSTM binding deficit that have so far been reported in AD patients – both sporadic cases and FAD – have been confined to tasks that probe colour-shape or colour–colour bindings (Parra et al., 2009, Parra et al., 2010, Parra et al., 2011). Such tasks are often considered to probe *conjunctive binding*: the ability to form a single representation of an item with multiple elements, with veridical retrieval depending crucially upon the ability to access the unitary, integrated representation (see Moses & Ryan, 2006). By contrast, retrieval of multi-feature items that can be performed by remembering individual parts separately (e.g., identity and location) is considered to depend upon relational binding (see Hannula et al., 2015). Whether the distinction between relational and conjunctive binding is a useful one is open to debate, but several studies have shown that conjunctive binding can be preserved in patients with hippocampal lesions (e.g., Baddeley, Allen, & Vargha-Khadem, 2010; Mayes et al., 2007; Parra et al., 2015).

These considerations therefore raise the possibility that the deficits in VSTM conjunctive binding reported in Alzheimer's cases (Parra et al., 2009, Parra et al., 2010, Parra et al., 2011) might not depend upon hippocampal loss. Furthermore, it has not been established whether relational binding deficits in VSTM occur in Alzheimer's disease, in addition to the conjunctive binding deficits that have already been documented. Finally, to date, there is no report of whether delayed reproduction tasks can also detect binding deficits in VSTM in AD. This might be important both for understanding normal hippocampal function and for early detection of MTL pathology, including Alzheimer's disease, since this type of task provides potentially sensitive measures compared to conventional ones, for example, those which reply on quantal measures such as span (Zokaei et al., 2014).

Here we test whether relational memory binding is impaired in a group of twenty individuals who were carriers of a genetic mutation known to be pathogenic for FAD using the ‘What was where?’ task which has previously detected pathological misbinding in patients with MTL lesions (Pertzov et al., 2013). Subsidiary analyses are performed in 12 asymptomatic cases and 8 symptomatic cases respectively in order to determine whether deficits can be detected in the asymptomatic group, i.e., before a formal diagnosis of dementia. To examine the relationship between performance on this task and the hippocampus, we also related misbinding rate to hippocampal volume. Object-location misbinding rate, using the same task parameters, does not increase with healthy ageing on this paradigm (Pertzov, Heider, Liang, & Husain, 2015). Thus detection of deficits using such a protocol might provide a useful means to detect pathologies which affect MTLs. Because of this practical consideration, one of our aims in this study on FAD is to establish the minimum number of trials required to demonstrate differences between cases and healthy controls.

## 2 Methods

### 2.1 Participants

Participants for the present study were recruited from an on-going longitudinal FAD study at the Dementia Research Centre, University College London (UCL), which receives referrals from across the UK. Individuals at risk of FAD were recruited into the study if there was an autosomal dominant family history of Alzheimer's disease and a known pathological mutation in either presenilin 1 (*PSEN1*) or amyloid precursor protein (*APP*) genes in at least one affected family member. Based on the results of the genetic tests and clinical assessments (see below), individuals were classified as symptomatic FAD individuals, asymptomatic FAD gene carriers or non-carriers.

*Symptomatic* individuals were those who had a positive genetic test and cognitive symptoms consistent with Alzheimer's disease. *Asymptomatic* gene carriers were at-risk individuals who had a positive genetic test but did not have symptoms and who scored zero on the Clinical Dementia Rating (CDR) (see below). Non-carriers were at-risk individuals who tested negative for pathological mutations. The controls for the study consisted of both non-carriers and healthy individuals recruited for the study. As the symptomatic and asymptomatic gene carrier groups differed significantly in terms of age, two different but overlapping sets of controls were selected from the entire control group (*n* = 62) to be age-matched for each gene carrier group (see Supplementary material: selection of control groups). Baseline characteristics of the groups are presented in Table 1 (section 3.1) and Supplementary Table 1.

All participants had normal or corrected-to-normal visual acuity and colour vision by self-report or according to their informants. We used “years from parental age of onset” as an indicator of how far the asymptomatic gene carriers were likely to be from manifesting symptoms (Bateman et al., 2012). This was calculated by subtracting the individuals' age at the time of the assessment from that at which their parents first developed symptoms of FAD (see Table 1). One symptomatic FAD individual was on acetylcholinesterase inhibitor treatment at the time of assessment. To ensure that level of performance was sufficiently above chance, we set a predefined minimum of 70% average accuracy in identification performance as an inclusion criterion (see section 2.4 VSTM experiment). On this basis, six symptomatic FAD participants who took part were excluded. The study was approved by the local ethics committees (University College London and University College Hospital London) and all subjects gave written informed consent.

### 2.2 Protocol

The study protocol included a clinical assessment, a neuropsychological assessment, the ‘What was where?’ VSTM experiment and a 3T structural MRI scan. Detailed interviews were conducted with individuals at risk of FAD and their close informants by a neurologist (YL, NF) to probe the presence of cognitive or behavioural symptoms attributable to Alzheimer's disease. Alzheimer's disease was diagnosed using the most up-to-date research criteria at the time of assessment (Dubois et al., 2007, Dubois et al., 2010). Folstein's mini-mental state examination (MMSE) (Folstein, Folstein, & McHugh, 1975), the CDR (Morris, 1993) and Hospital Anxiety and Depression scale (HADS) (Zigmond & Snaith, 1983) were administered to all participants. Genetic results were available for all at-risk individuals, either on a clinical or research basis. Research genetic results were only fed back to the statistician involved in the study and were not disclosed to the participants or to other researchers.

### 2.3 Neuropsychological assessment and statistical analysis

The neuropsychological test battery included the Recognition Memory Test for words and faces (RMT words and faces) (Warrington, 1984), story recall from the logical memory subset of Wechsler Memory Scale-Revised (WMS-logical memory) (Wechsler, 1987), Rey complex figure (measured as the ratio of score for the immediate delay condition over score for the copy condition) (Osterreith, 1944), digit span (Wechsler, 1987) and spatial span (Kessels, van Zandvoort, Postma, Kappele, & de Hann, 2000). Measures of current intelligence, executive function, confrontational naming, arithmetic, visual perception, speed and estimate of premorbid intelligence (the National Adult Reading Test) (NART) (Law and O'Carroll, 1998, Nelson, 1983) were also included (see Supplementary material: Neuropsychology tests).

Linear regression was used to compare neuropsychological test scores between the entire FAD group and controls, the symptomatic group and age-matched controls and between the asymptomatic group and their controls. Where test scores were not normally distributed, we transformed the data where suitable approximations to the normal distribution could be achieved. Where parametric assumptions were not met even after transformation, analysis proceeded with the untransformed score and bias-corrected accelerated bootstrap confidence intervals for the differences between groups were provided based on 2000 replications. All comparisons were adjusted for the effects of NART and sex.

### 2.4 VSTM experiment

**Fig. 1:**
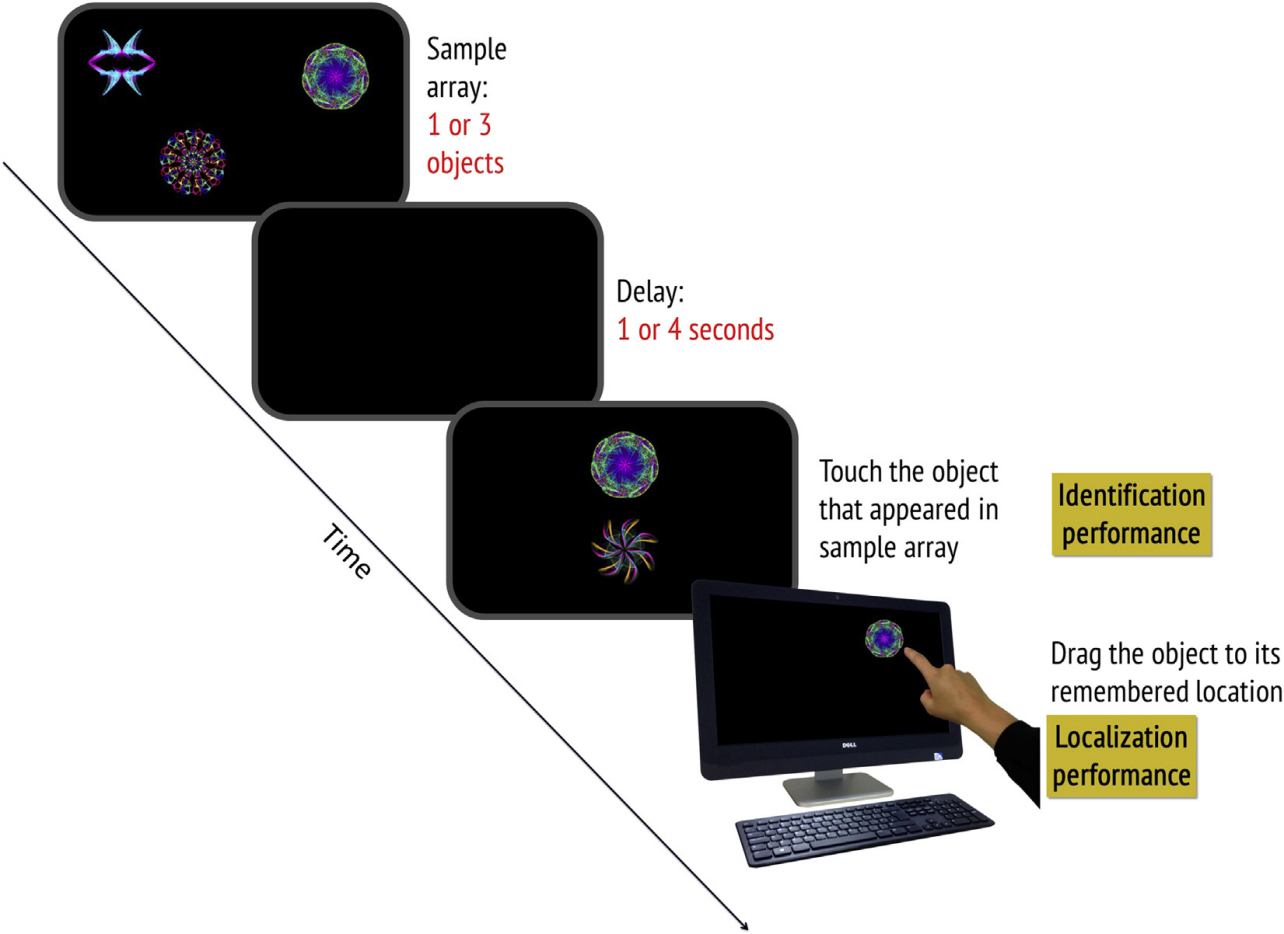
**Schematic of ‘What was where?’ task** One or three fractals were shown prior to a variable delay of either 1 or 4 sec, after which one of the objects was displayed together with a foil (distractor which had not appeared in the memory array). Participants were required to touch the item they recalled (**identification performance**) and drag it to its remembered location (**localization performance**).

The stimuli and procedure used have been described in detail in previous manuscripts (Pertzov et al., 2012, Pertzov et al., 2013, Pertzov et al., 2015). A schematic of the task is shown in Fig. 1. Participants sat approximately 42 cm in front of an interactive touch-sensitive screen (Dell Inspiron One 2320) with a 1920 × 1080 pixel matrix corresponding to approximately 62 × 35° of visual angle. In each trial, participants viewed 1 or 3 fractal objects, each randomly located on the screen. They were asked to remember both the objects and their locations. A blank screen was then displayed for 1 or 4 sec duration, followed by a test array in which two fractals appeared along the vertical meridian. One of these was in the memory array, which we call the *target fractal* whereas the other one was a foil or distractor. The foil was not an unfamiliar object, but was part of the general pool of fractal images presented across the experiment.

Participants were required to touch the fractal which they remembered to have been in the memory array and drag it on the touch screen to its remembered location. This provides us with a continuous, analogue measure of localization error. Each participant performed a practice block of 10 trials followed by two test blocks. Each test block consisted of ten trials with one fractal and 40 trials with three fractals. In each test block, the number of trials with one or three fractals and 1 sec or 4 sec delay between memory and test arrays were balanced.

Fractal stimuli were drawn from a library of 60 pictures of fractals (see Supplementary Fig 1; http://sprott.physics.wisc.edu/fractals.htm). Each fractal was presented between 2 and 3 times in different trials within the block. The locations of the fractals were determined by a Matlab script (MathWorks, Inc) in a pseudorandom manner, with several restrictions.

Importantly, fractals were never located within 9° of each other in order to prevent spatial uncertainty as a result of crowding and to create a clear zone around the original locations of the items which is critical for the analysis of localization errors. Moreover they were positioned with a minimum of 3.9° from the edges of the screen and 6.5° from the centre of screen.

Memory for **object identity** was measured as the proportion of trials where the correct object was chosen in the test array. **Gross localization error** was computed as the distance (expressed as visual angle) between the centre of the target object after it had been dragged to its remembered location and its true (original) location in the memory array. It was only measured on trials where an object was correctly identified.

Previous studies have indicated that when participants mislocalize objects, some of their reports can be clustered around the locations of other objects in the memory array, rather than occurring randomly (Pertzov et al., 2012, Pertzov et al., 2013). We call these **swap errors** because the location of the target fractal was swapped with that of another fractal in the original memory array. The number of swap errors was indexed by the percentage of correctly identified objects placed within 4.5° eccentricity of other fractals in the original array. As in previous studies, we used a threshold of 4.5° because objects were never presented less than 9° from each other in the memory array. Using a cut-off of 4.5° means that the reported location of an object could never be attributed to more than one object.

It might be argued that objects localized further away from their original location simply by chance might lead to more apparent swap errors. To ensure that swap errors did not simply result from increased gross localization errors, we also used a measure of **swap errors corrected for chance** (see Supplementary material for calculation as originally described (Pertzov et al., 2013)).

What effect do swap errors have on the overall gross localization error? Can they explain all of the memory deficits in remembering the location of the target fractal? To answer these questions, crucially we also computed the distance between the remembered location of the target fractal and the nearest fractal in the original memory array, *regardless* of whether it was the target. This **nearest neighbour control** analysis provides a simple index of the **localization precision** regardless of object identity. It effectively provides a measure of localization error subtracting out the effects of swap errors (see Pertzov et al., 2013). Comparison of gross localization error with the error computed by the nearest neighbour control analysis therefore provides an important measure of the impact of swap errors on overall recall localization. For detailed description of the statistical analysis of VSTM outcomes, see Supplementary material: Statistical analysis for VSTM outcomes.

### 2.5 Brain image acquisition and statistical analysis of relationships between VSTM outcomes and hippocampal volumes

T1-weighted volumetric MR brain images were acquired on a 3T Siemens TIM Trio scanner using a magnetisation prepared rapid gradient echo (MPRAGE) protocol acquired in sagittal orientation (TR = 220 msec, TE = 2.9 msec, TI = 900 msec, Flip angle = 9°, FOV = 282 × 282 × 228 mm, voxel size = 1.1 × 1.1 × 1.1 mm). Hippocampal volumes were estimated using a template-based method for automated segmentations (Jorge Cardoso et al., 2013) and manually edited where required. For each participant, total hippocampal volume (sum of left and right hippocampus) was calculated. We generated a head size measure by estimating total intracranial volumes (TIV) from the summation of the volumes of grey matter, white matter and cerebral spinal fluid using the segmentation toolbox in Statistical Parametric Mapping version 8 (Friston, Ashburner, Kiebel, Nicholas, & Penny, 2007; Leung et al., 2010).

Linear regression was used to compare hippocampal volume between groups, adjusting for age, sex and TIV. To examine the association between hippocampal volume and the outcomes of the VSTM task (overall memory for object identity and localization and overall swap error rates), we used the same modelling approach as described for analysis of VSTM outcomes (see Supplementary material statistical analysis for VSTM outcomes). The analyses compared the association between hippocampal volume and VSTM outcomes between the entire FAD group and controls, by including main effects for group, hippocampal volume (entered as a continuous predictor), and interaction between hippocampal volume and group. To ensure that any association found in FAD participants was not simply driven by differences in hippocampal volume between asymptomatic and symptomatic gene carriers, analyses were then repeated with inclusion of separate terms for these two groups and their interactions with hippocampal volume. All analyses were adjusted for age, sex and TIV. Supplementary material describes statistical methods in relation to associations between hippocampal volumes and neuropsychological measures.

## 3 Results

### 3.1 Baseline characteristics of participants

Asymptomatic FAD gene carriers had similar baseline characteristic as age-matched controls except for slightly lower HAD depression score and fewer years of formal education. As expected, symptomatic gene carriers had lower MMSE and NART scores than age-matched controls (Table 1). See also Supplementary Table 1 for the results of the entire FAD group.

### 3.2 Neuropsychological assessment

Asymptomatic gene carriers were not, on average, significantly different to their controls in any of the measures including conventional indices of WM (e.g., digit and spatial spans), other than WASI IQ score (controls = 116.9, asymptomatic = 103.6, *p* < .001) (see Table 2). On the other hand, symptomatic FAD individuals were, on average, significantly worse than their controls on IQ, RMT for words, WMS-logical memory immediate and delayed conditions, Rey complex figure, digit span backward maximum, spatial span forward maximum, Stroop test, Trail making, Graded Difficulty Arithmetic (GDA) test and the digit symbol test (see Table 2). See also Supplementary Table 2 for results of the entire FAD group.

### 3.3 VSTM experiment

#### 3.3.1 *All FAD cases*

Consistent with previous studies (Pertzov et al., 2015, Pertzov et al., 2012), performance was significantly influenced by memory load (1 or 3 objects), delay (1 or 4 sec) and block (first *vs* second block of trials) for both object identification and gross mislocalization error such that all participants (FAD cases and controls) were worse in higher memory load and longer delays and improved in the second block.

The FAD group performed significantly worse than controls in memory for object identity (FAD = 86.7% *vs* controls = 91.7%, *p* = .009, *z* = −2.61) as well as in gross localization memory performance, measured as raw error from the original location of the probed item in the memory array (FAD = 7.89° *vs* controls = 5.64°, *p* = .001, *t* = 3.39).

**Fig. 2:**
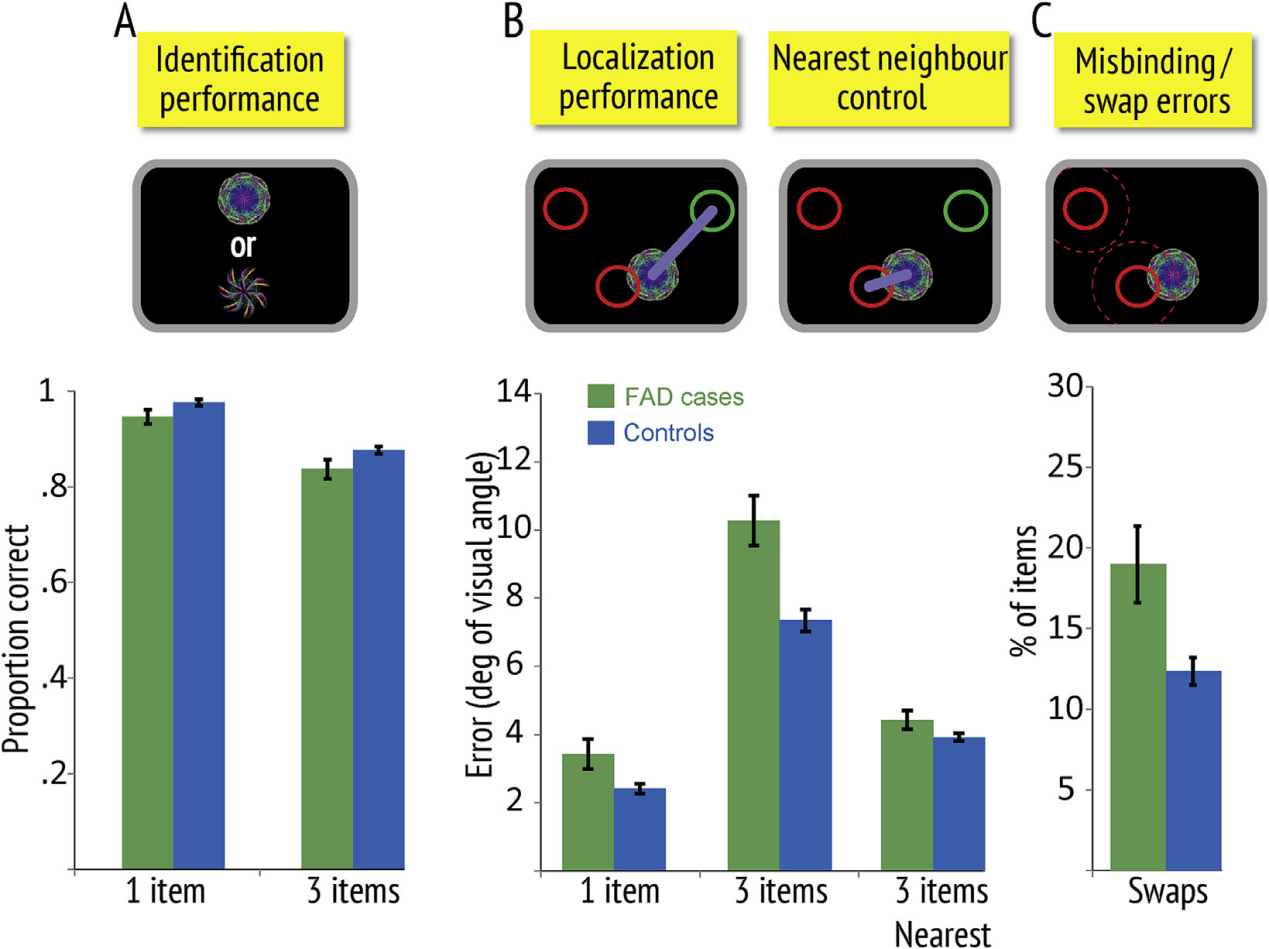
Memory performance of all FAD cases versus controls in first block. **(A) Identification performance** for one or 3 items in the memory array. **(B) Localization performance** (gross localization error) – measured as error from the true location of the item in the memory array. The “nearest neighbour” control error was calculated as the minimal distance between a reported location and any one of the previously presented fractals for three-item trials. Top inset images illustrate how outcomes are measured. Circles represent the original location of the target fractal (green) and two other, non-probed fractals (red); purple lines illustrate how localization errors are measured for gross localization and nearest neighbour distances. **(C) Swap or misbinding errors** are proportion of times target objects were localized close to the remembered locations of *non-probed fractals* in the original display (red circles). The inset image above shows how a target fractal might be misplaced to the location of a non-probed item, thereby generating a swap error. Error bars represent standard errors of the mean.

For localization, there was a significant interaction between group and block, as well as a significant triple interaction between group, block and item number [*F*(3,81) = 4.79, *p* = .004]. Further analysis revealed that FAD participants were significantly impaired in both the 1- and 3-item conditions in the first block (Fig. 2), but in the second block this was the case for only the 3-item condition (*Block 1 for 1 item*: FAD = 3.44° *vs* controls = 2.42°, *p* = .009, *t* = 2.67; *Block 1 for 3 items*: FAD = 10.2° *vs* controls = 7.28°, *p* < .001, *t* = 3.99; *Block 2 for 1 item*: FAD = 2.43° *vs* controls = 2.33°, *p* = .41, *t* = .83; *Block 2 for 3 items*: FAD = 7.59° *vs* controls = 5.63°, *p* = .009, *t* = 2.69).

Thus, just as observed in healthy controls on this task (Pertzov et al., 2015), there was evidence of learning across blocks, with the biggest difference between FAD individuals and controls apparent in the first block. Importantly, for practical purposes, this finding demonstrates that testing confined to only 50 trials is sufficient to distinguish FAD cases from controls.

When they correctly identified the objects, FAD individuals were significantly more likely to make *swap errors* than controls (FAD = 16.5% *vs* controls = 10.6%, *p* = .006, *t* = 2.84). Thus they mislocalized the probed item to the position of another object in the original memory array more often than healthy controls. Even after controlling for swap errors due to chance (see Methods 2.4), the group difference remained significant (FAD = 11.2% *vs* controls = 7.12%, *p* = .006, *t* = 2.83). In the first block alone, the FAD group also made significantly more errors than controls (FAD = 18.9% *vs* controls = 12.3%, *p* = .005, *t* = 2.85). A main effect of block was found, reflecting lower number of swaps in the second block in both groups.

As it has previously been shown that healthy participants make significantly more swap errors when delay length is extended (Pertzov et al., 2012), we also examined the effect of delay on swap errors (see Methods 2.4) and found a borderline significant group and delay interaction (*p* = .08, *t* = −1.79). Further analysis revealed that the FAD group was significantly worse than controls in the longer delay condition (FAD = 18.6% *vs* controls = 10.7%, *p* = .002, *t* = 3.25) but not over shorter delays (FAD = 14.4% *vs* controls = 10.6%, *p* = .13, *t* = 1.54).

These analyses show that overall the FAD group was significantly more likely to misbind identity and location of items, and this was detectable with just one block of trials, with the longer delay more likely to reveal greater misbinding. But do swap errors explain all the error on localization memory performance? To examine this we next performed the “nearest neighbour control” analysis which determines localization error with respect to the nearest item in the original memory array i.e., localization precision, *regardless* of whether this was the correct location of the item probed (see Methods 2.4). This allowed us to establish whether the additional error in mislocalization observed in the FAD group could be entirely attributed to swap errors, in which case there would be no significant difference between the groups on localization error computed with respect to the nearest neighbour.

When localization error was measured with respect to the nearest neighbour in the memory array, the difference between FAD cases and controls reduced considerably, indicating that misbinding errors made a large contribution to their gross localization error. However, there still remained a significant difference between the groups (overall FAD = 4.30° *vs* controls = 3.69°, *p* = .012, *t* = 2.58; in first block FAD = 4.41° *vs* controls = 3.86°, *p* = .049, *t* = 2.00; Fig. 2). Therefore, in addition to making significantly more swap errors, there was an extra source of error in the overall FAD group. This source of localization error might be due to noisier encoding, storage, recall or all three of these potential processes. The crucial point is that the delayed VSTM reproduction task was able to show deficits in the FAD group overall, both in memory for identity and location. Furthermore, location memory over a few seconds was significantly corrupted by misbinding errors, but these did not account completely for all the gross localization error.

#### 3.3.2 *Asymptomatic* gene carriers

Next, we examined the performance of only asymptomatic gene carriers. Compared to age-matched controls, across the two blocks, they did *not* differ significantly in their ability to remember the identity of the fractals (asymptomatic FAD = 89.9% *vs* controls = 92.1%, *p* = .29, *z* = −1.06) or in gross localization error (asymptomatic FAD = 6.47° *vs* controls = 5.58°, *p* = .12, *t* = 1.58). Both groups showed learning across blocks and worse performance with longer delay and higher memory load. Critically, as previously, there was a significant group by block interaction in *localization performance* (*p* = .03, *t* = −2.27).

**Fig. 3:**
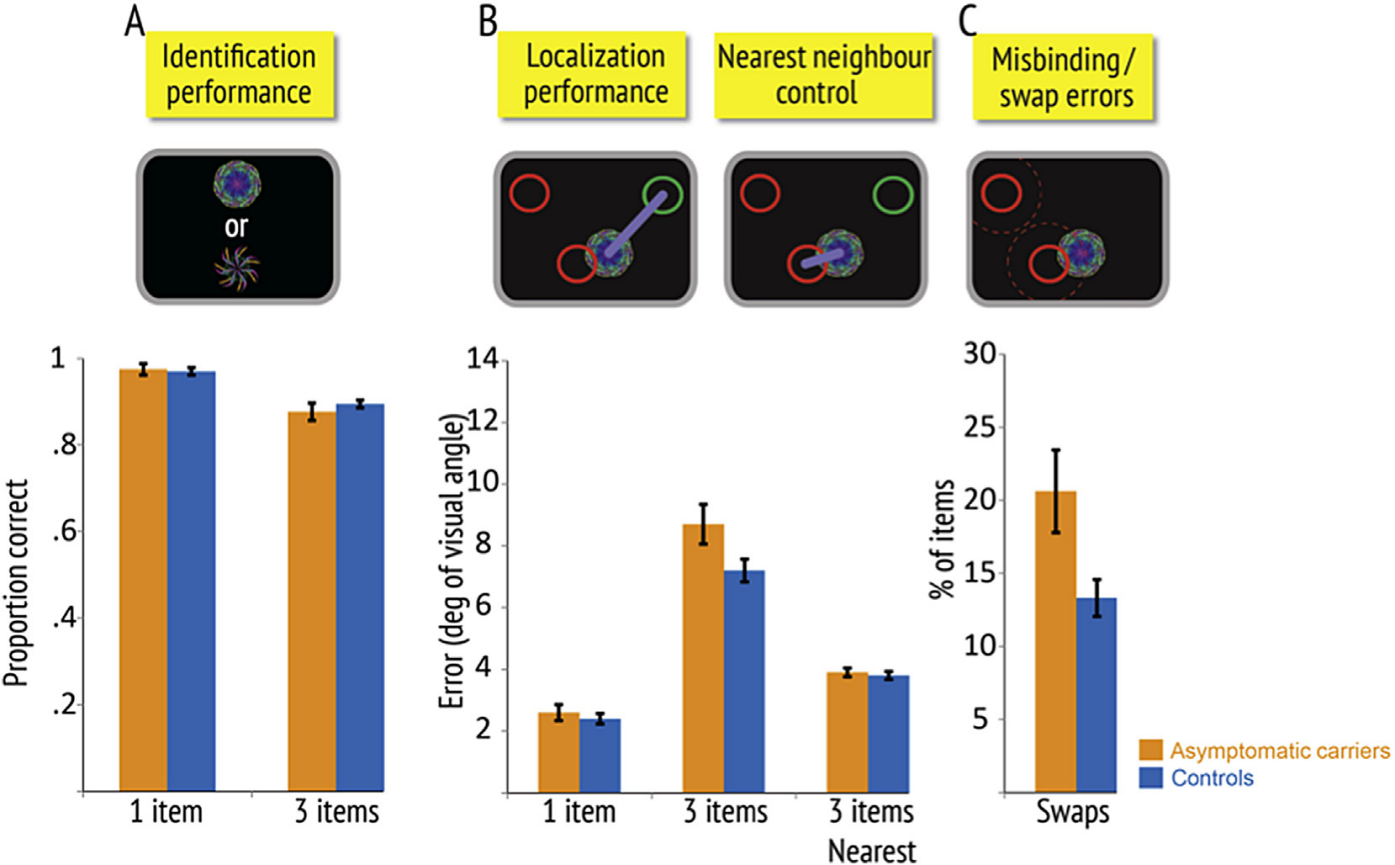
Memory performance of asymptomatic carriers versus controls in first block. **(A) Identification performance:** proportion of times participants selected the correct fractal on two-alternative forced choice, when there were one or three items in the memory array. **(B) Localization performance** shows gross localization error – simply measured as the error from the true location of the item in the memory array. The “nearest neighbour” control error (localization precision) was calculated as the minimal distance between a reported location and any one of the previously presented fractals for three-item trials. Top inset images illustrate how the outcomes are measured. Circles represent the original location of the target fractal (green) and two other, non-probed fractals (red); purple lines illustrate the localization errors for the two different measures. **(C) Swap or misbinding errors 4 sec delay**: proportion of times target objects were localized close to the remembered locations of non-probed fractals in the original display (red circles). The inset image above shows how a probed fractal might be misplaced to the location of one of the *non-probed items*, thereby generating a swap error. Error bars represent standard errors of the mean.

Assessment of the data of each block separately revealed that while asymptomatic gene carriers were significantly worse in localization memory than controls in the first block (asymptomatic FAD = 7.52° *vs* controls = 6.25°, *p* = .03, *t* = 2.19), there was no difference in the second block (asymptomatic FAD = 5.42° *vs* controls = 4.90°, *p* = .40, *t* = .84). Thus, once again, the biggest difference from controls was apparent with only one block of testing (Fig. 3). Further analysis revealed that asymptomatic gene carriers were significantly worse than controls in only the multiple item conditions in the first block (*3 items*: asymptomatic FAD = 8.74° *vs* controls = 7.21°, *p* = .02, *t* = 2.33; *1 item*: asymptomatic FAD = 2.65° *vs* controls = 2.38°, *p* = .16, *t* = 1.43; Fig. 3). Note that swap or misbinding errors, by definition, can of course only occur when there is more than one item to remember.

To evaluate the contribution of misbinding to the impairment in localization memory in this condition, we computed the frequency of swap errors. As delay has an effect on swap errors in the entire FAD group, we also examined the effect of delay and block on swap errors here. There was a borderline significant three-way interaction between group, block and delay [*F*(3, 61) = 2.54, *p* = .06]. Compared to controls, asymptomatic gene carriers made significantly more swap errors in the 4 sec delay condition of the first block (Fig. 3: asymptomatic FAD = 20.6% *vs* controls = 13.3%, *p* = .009, *t* = 2.71). This was evident even after controlling for swap errors due to chance (asymptomatic FAD = 13.6% *vs* controls = 9.1%, *p* = .03, *t* = 2.24). Thus the asymptomatic carriers group was significantly more likely to misbind identity and location of items in the longer delay condition.

As with the analysis for the FAD group overall, we next investigated whether all the error in localization performance of asymptomatic gene carriers could be attributed to identity-location misbinding. To do so, we used the “nearest neighbour control” analysis to measure localization precision as before. Critically, when this was performed, the difference in localization memory performance between asymptomatic gene carriers and controls in the extended delay condition of the first block was no longer significant (Fig. 3: asymptomatic FAD = 3.92° *vs* controls = 3.83°, *p* = .55, *t* = .38). This finding strongly suggests that the increased gross mislocalization error of asymptomatic FAD cases can be accounted for entirely by their increased tendency to make swap errors, i.e., misbinding item identity and location.

In summary, the asymptomatic gene carriers were significantly worse than controls in localization memory performance in the first block when multiple items were remembered. This deficit can be attributed specifically to increased swap errors when there was longer delay between the memory and test conditions and not impaired precision of localization *per se*, e.g., due to increased noise in memory. Thus recall in these individuals seems to be systematically corrupted by interference from other items in memory. Note that this is unlike the analysis for location memory for all FAD cases reported above which cannot entirely be attributed to misbinding errors alone.

#### 3.3.3 *Symptomatic* FAD cases

Unlike asymptomatic gene carriers, symptomatic FAD cases were overall significantly worse than age-matched controls both in their ability to remember object identity (symptomatic FAD = 81.8% *vs* controls 91.3%, *p* < .001, *z* = −4.71) and location (symptomatic FAD = 10.0° *vs* controls = 5.90°, *p* < .001, *t* = 4.76). For gross localization, there was a significant interaction between group and block, as well as a significant three-way interaction between group, item and block [*F*(2,35) = 6.88, *p* = .003].

**Fig. 4:**
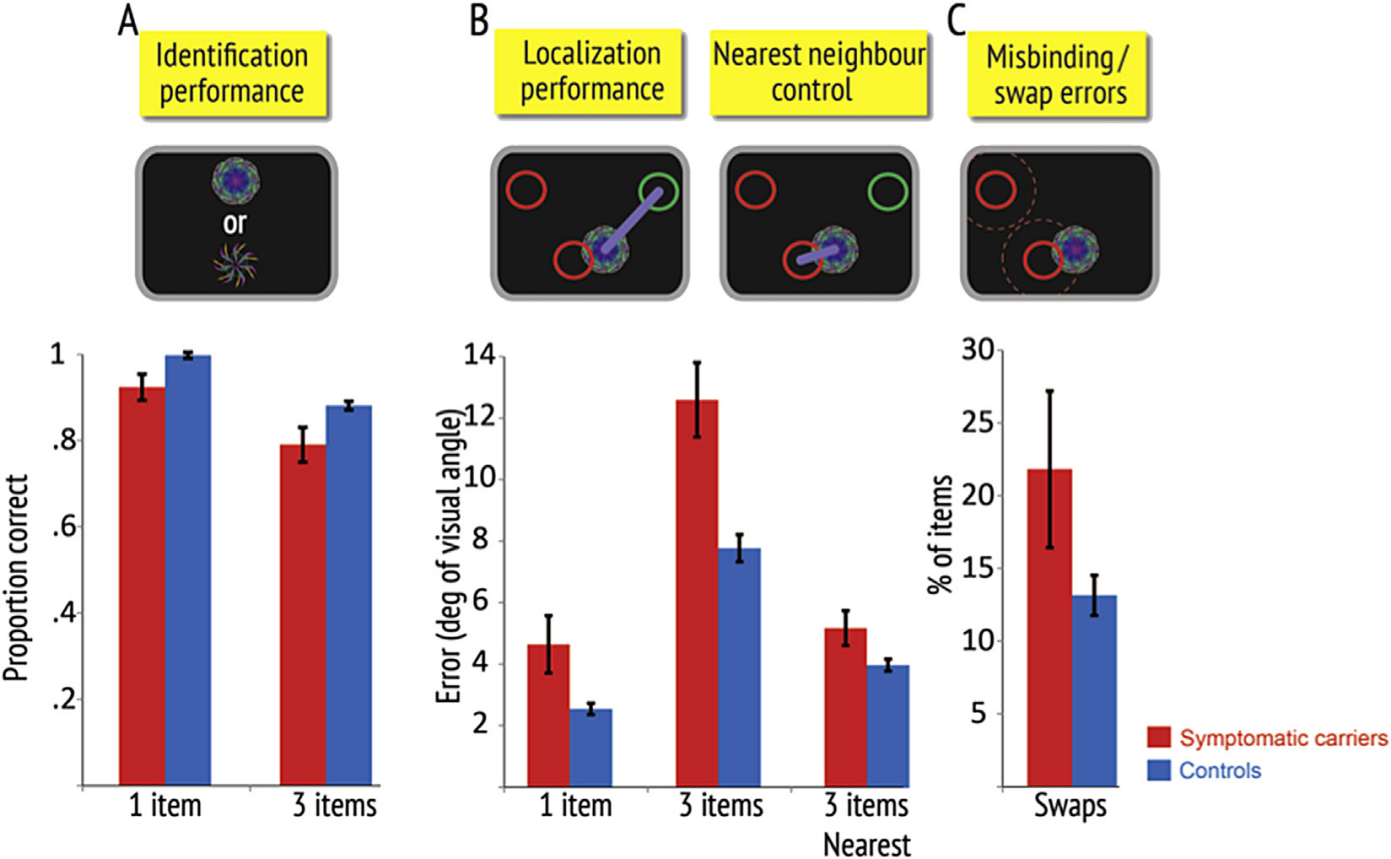
Memory performance of symptomatic FAD cases versus controls in first block. **(A) Identification performance** for one or three items in the memory array. **(B) Localization performance** (gross localization error) – measured as error from the true location of the item in the memory array. The “nearest neighbour” control error was calculated as the minimal distance between a reported location and any one of the previously presented fractals for three-item trials. Top inset images illustrate how the outcomes are measured. Circles represent the original location of the target fractal (green) and two other, non-probed fractals (red); blue lines illustrate the localization errors for the two different measures. **(C) Swap or misbinding errors** are proportion of times target objects were localized close to the remembered locations of non-probed fractals in the original display (red circles). The inset image above shows how a target fractal might be misplaced to the location of a non-probed item, thereby generating a swap error. Error bars represent standard errors of the mean.

Thus symptomatic FAD individuals were significantly worse than controls in *both* 1- and 3-item conditions in the first block (Fig. 4: *1 item*: symptomatic FAD = 4.62° *vs* controls = 2.54°, *p* = 0.01, *t* = 2.58; *3 items*: symptomatic FAD = 12.5° *vs* controls = 7.72°, *p* < .001, *t* = 4.18). Note that this differs from asymptomatic gene carriers who were only impaired on the 3-item condition in the first block. Symptomatic cases, like healthy controls and asymptomatic gene carriers, showed learning (see Pertzov et al. 2015 for a detailed discussion on learning in healthy participants). Thus, in the second block the difference between them and controls was apparent only for 3-items trials (*1 item*: symptomatic FAD = 2.68° *vs* controls 2.30°, *p* = 0.24, *t* = 1.20; *3 items*: symptomatic FAD = 9.97° *vs* controls = 5.82°, *p* < .001, *t* = 5.52). Again, this differs from asymptomatic gene carriers who were not significantly different from healthy controls in the second block.

Next, we assessed the contribution of swap errors to the impairment in the localization memory. Symptomatic FAD cases made significantly more swap errors than controls overall (symptomatic FAD = 21.3% *vs* controls = 11.6%, *p* < .001, *t* = 4.12). Even after controlling for swap errors due to chance, the overall group difference remained significant (symptomatic FAD = 14.3% *vs* controls = 7.8%, *p* = .008, *t* = 2.82). Symptomatic FAD cases also made significantly more errors than controls in the first block (Fig. 4: symptomatic FAD = 21.6% *vs* controls = 13.0%, *p* < .05, *t* = 2.06). However, there were no significant two-way interactions between group and delay, or three-way interactions between group, delay and block. Thus the symptomatic FAD group was significantly more likely to misbind identity and location of items. But does this explain all their error on localization memory performance, just as it did for asymptomatic cases?

We again used the “nearest neighbour control” analysis to investigate this. Unlike asymptomatic gene carriers, symptomatic FAD cases remained significantly impaired compared to controls on this purer localization precision measure too, both overall and in the first block (Fig. 4: *Block 1*: symptomatic FAD = 5.14° *vs* controls = 3.95°, *p* = .009, *t* = 2.44; *Overall*: symptomatic FAD = 4.82° *vs* controls = 3.73°, *p* = .005, *t* = 3.00). Thus their poor memory for location cannot be attributed solely to increased misbinding of identity to location.

In summary, the symptomatic FAD group was significantly impaired in memory for object identity and gross localization for the 3-item condition. Unlike asymptomatic cases, their increased gross mislocalization was due to *both* increased swap errors (misbinding) and reduced precision of localization. Degradation of localization precision was also evident in localization errors even when they had to remember one item (i.e., when no misbinding was possible), at least in the first block.

### 3.4 Hippocampal volumes and correlations with VSTM outcomes

54 controls, 12 asymptomatic and six symptomatic gene carriers had usable structural MRI scans. Mean (SD) total (left plus right) raw hippocampal volumes in these groups were 5.8 (.64), 6.0 (.69) and 5.2 (.55) cm^3^ respectively.

After adjusting for the effects of age, sex and TIV, the hippocampal volumes of the asymptomatic gene carriers were not significantly different to the control volumes (mean difference .26 cm^3^, *p* = .10). However, symptomatic individuals had significantly smaller hippocampal volumes compared with both controls (mean difference .67 cm^3^, *p* = .003) and asymptomatic gene carriers (mean difference .93 cm^3^, *p* = .001).

There was no statistically significant association between identification performance and hippocampal volumes in either the entire controls group (odds ratio = .94, *p* = .64) or the entire FAD group (odds ratio = 1.35, *p* = .15) without any significant interactions between the groups (odds ratio = 1.44, *p* = .10).

**Fig. 5:**
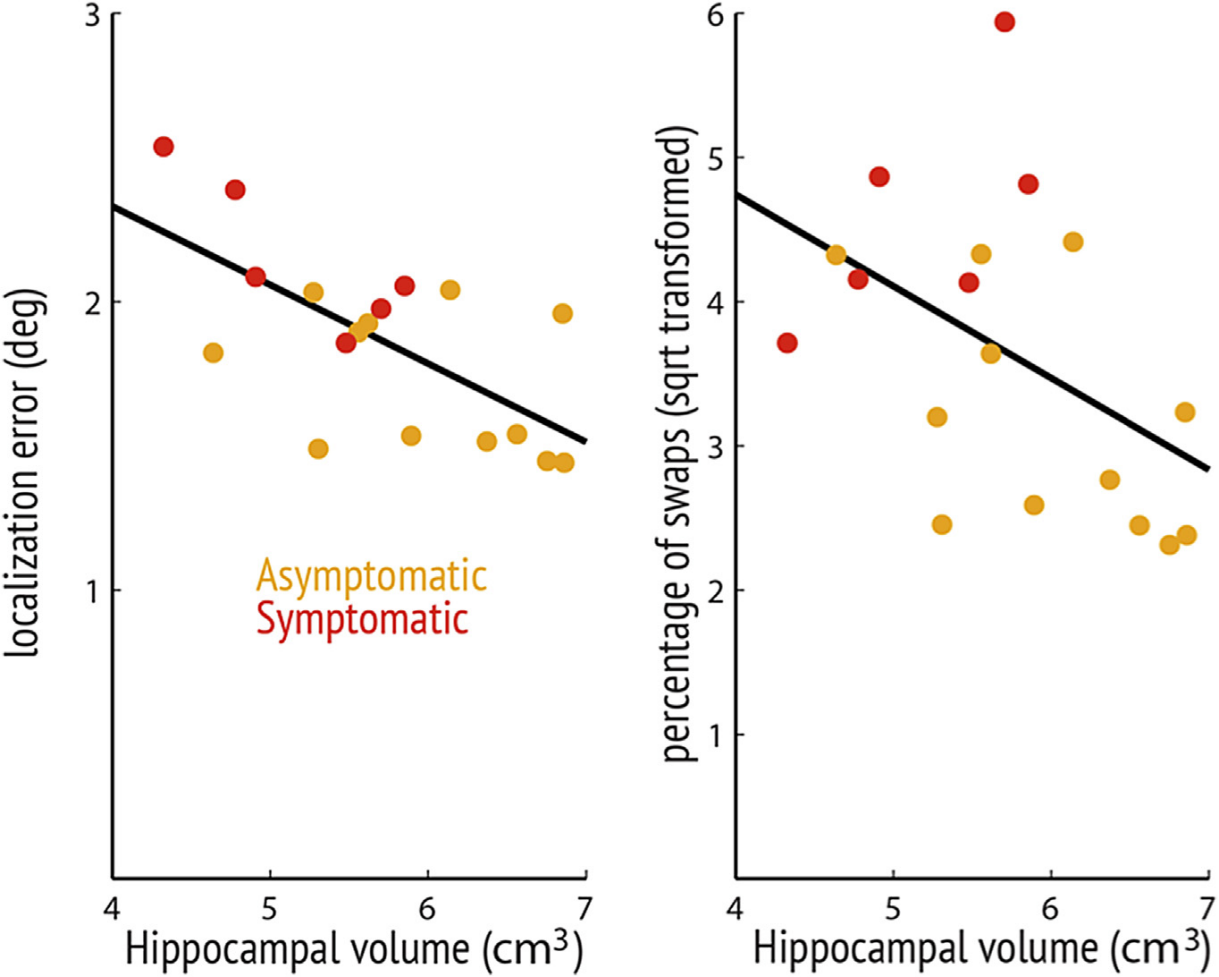
Relationship between hippocampal volume and memory. Total hippocampal volumes (adjusted for TIV) were inversely correlated with overall gross mislocalization error and overall swap errors (square root transformed) across FAD individuals.

Unlike identification performance, there was a statistically significant association between gross mislocalization error and total hippocampal volume in the entire FAD group (21% reduction in error per cm^3^ increase in volume, *p* = .02) (Fig. 5) but not in controls (2% reduction per cm^3^, *p* = .79) and the group interaction was marginally significant (mean difference 19% reduction per cm^3^, *p* = .050). The association in the FAD group appeared to be driven by symptomatic (41% reduction per cm^3^, *p* < .001) rather than asymptomatic gene carriers (7% reduction per cm^3^, *p* = .42) with significant interactions between both symptomatic individuals and controls (mean difference of 42% reduction per cm^3^, *p* < .001) and between symptomatic and asymptomatic gene carriers (mean difference of 37% reduction per cm^3^, *p* = .003). However, there were no significant associations between hippocampal volume and pure localization precision (as measured using the “nearest neighbour control” analysis) in either the entire FAD group (7% reduction per cm^3^, *p* = .35) or controls (1% reduction per cm^3^, *p* = .82) with no interaction between the two groups (mean difference of 11% reduction per cm^3^, *p* = .21). This suggests that hippocampal volume was more likely to be associated with swap errors rather than localization precision *per se*.

Lastly, there was a significant association between proportion of overall swap errors and hippocampal volume in the entire FAD group (regression coefficient = −.76, *p* < .001) (Fig. 5) but not in controls (regression coefficient = −.03, *p* = .91) with significant interaction between the two groups (mean difference in regression coefficient = −.73, *p* = .008). The correlation in the FAD cases is significant even when considering only asymptomatic gene carriers (regression coefficient = −.64, *p* = .045) but not in the symptomatic cases (regression coefficient = .71, *p* = .15). There were significant interactions between the asymptomatic gene carriers and controls (mean difference in regression coefficient = −.68, *p* = .02) and between asymptomatic and symptomatic gene carriers (mean difference in regression coefficient = 1.35, *p* = .02). See Supplementary material for association between neuropsychology tests and hippocampal volumes.

### 3.5 Relationship between depression (HAD) scores and swap error rate

There was no statistically significant association between HAD depression scores and the average misbinding rate (swap error rate) in either the FAD cohort as a whole (coefficient = −.01, *p* = .93) or in controls (coefficient = .02, *p* = .76) using regression analysis with no statistically significant interaction between the two groups (coefficient = .02, *p* = .82).

## 4 Discussion

VSTM in individuals with pathological mutations for FAD was investigated using a recently established, delayed reproduction paradigm that allows assessment of participants' recognition memory for object identity independent of recall of its location (Pertzov et al., 2012, Pertzov et al., 2013). By using a continuous scale for report of object location, it was possible to probe not only the magnitude but also the nature of localization errors. Overall, FAD mutation carriers showed significantly worse memory for both object identity and location. Crucially, they more frequently mislocalized the probed item (target fractal) to the location of one of the other, non-probed fractals held in memory array (Fig. 2). Such swap or relational *binding errors* provide direct behavioural evidence of an impaired ability to bind together memory for object identity to its location.

For the entire FAD group, misbinding of object identity and location accounted for much of their mislocalization error, but not for all of it. In the *asymptomatic* gene carriers, however, this was the only deficit identified when multiple objects were present in the memory array for 4 sec, accounting fully for the localization deficit in these individuals (Fig. 3). Thus their impairment in recalling the location of the probed item was systematically corrupted only by the locations of other items in the memory array. As this was only evident in the longer delay condition, it suggests that the impairment may be related to difficulty in maintenance processes rather than memory encoding or retrieval as impairment in these processes should influence performance in the short delay as well. Furthermore, it was observed only in the first block of the experiment. This may reflect the ability of participants to successfully recruit high level strategies leading to significantly improved performance with practice (Pertzov et al., 2015). The learning effect could explain why differences in relational binding performance between asymptomatic mutation carriers and controls was observed only in the most challenging condition, i.e., longer delay condition in the first block.

These misbinding errors cannot be explained by a failure to remember the identity of the objects as *asymptomatic* gene carriers exhibited normal performance when required to recognize fractals in the memory array and localization analysis was performed only in trials with accurate identification. Furthermore, the “nearest neighbour control” analysis – which measures the shortest distance from any fractal in the original memory array to the location where the probed item was located by the participant – shows that they also remembered the locations of the fractals well (Fig. 3). This points to the conclusion that although the locations of items in the memory array were retained in asymptomatic gene carriers, they were not correctly bound to the identities of the fractals that occupied those locations – a deficit of *relational binding* (Eichenbaum, 2006, Konkel et al., 2008, Mayes et al., 2007).

This finding echoes directly the recently-reported similar result in VGKC-Ab mediated limbic encephalitis using exactly the same paradigm (Pertzov et al., 2013). Because both FAD cases and VGKC-Ab patients have evidence of hippocampal atrophy or lesions respectively (Fox et al., 1996, Fox et al., 1996, Khan et al., 2009, Pertzov et al., 2013, Ridha et al., 2006, Schott et al., 2003), there is now compelling convergent evidence of a role for the hippocampus in relational binding even over short retention delays.

*Symptomatic* FAD cases in the current study also showed increased swap errors. In addition, they also had deficits in memory for individual features, namely, object identity and location even for 1 item (Fig. 4), where there is obviously no scope for an object-location misbinding error.

For all FAD cases, there was a significant negative correlation between hippocampal volume and swap error rate (Fig. 5), but not for object identity or localization *per se*, again consistent with the view of a strong relationship between hippocampus and relational binding. The lack of a significant correlation between hippocampal volume and swap errors in the symptomatic group may be due to their exaggerated localization error so even when they misremembered the location of a fractal to that of another fractal, their localization was too imprecise for it to count as a swap error (above the threshold or outside the perimeter we used to define mislocalization to another item in the array). In other words: to count as a swap error, the fractal needs to be precisely localized at the location of one of the non-target items. If localization precision is generally poor, such as in the symptomatic cases, our method would be expected to miss a fraction of swap errors. Misbinding error also does not appear to be related to depression, as evidenced by a lack of correlation between the HAD depression score and swap error rate (see Results section 3.5).

The results presented here also show that in FAD, object-location misbinding errors are observable with just one block of 50 trials, even when performance on standard neuropsychological tests of WM and long-term memory did not differ from healthy controls. These findings extend emerging reports on VSTM in Alzheimer's disease which have documented deficits in *conjunctive binding*, for colour-shape or colour-colour, before deficits on other tests are apparent (Parra et al., 2009, Parra et al., 2010, Parra et al., 2011). The hippocampal literature makes a distinction between relational and conjunctive binding (for a detailed comparison, see Moses & Ryan, 2006). Conjunctive binding refers to the ability to form a single representation of an item composed of several elements, with correct retrieval depending crucially upon the ability to access this unitary, integrated representation (see Moses & Ryan, 2006). According to this view, some have proposed that the hippocampus stores associations as well as the features. By contrast, for relational binding the hippocampus may store associations but not the features themselves, which may be retained in disparate cortical sites, e.g., for object identity and location.

While such a dichotomy is clearly open to debate, several investigators have swayed strongly towards the conclusion that the hippocampus is crucial for relational binding for long-term storage of items (Cohen and Eichenbaum, 1993, Eichenbaum, 2006, Konkel et al., 2008, Moses and Ryan, 2006), but is less critical for item memory or binding of features within objects (Baddeley et al., 2010; Konkel et al., 2008, Murray and Mishkin, 1998, Staresina and Davachi, 2008). Indeed, several studies have reported that conjunctive binding can be preserved in hippocampal patients (Baddeley et al., 2010; Mayes et al., 2007, Parra et al., 2015) and recent neurophysiological studies provide evidence that the hippocampus or MTL structures may act as a hub for integrating and co-ordinating disparate cortical representations to support relational binding (Cashdollar, Duncan, & Duzel, 2011; Watrous, Tandon, Connor, Pieters, & Ekstrom, 2013).

The findings presented here and previously in VGKC-Ab cases (Pertzov et al., 2013) suggest that the relational binding role of the hippocampus is not confined to long-term memory but also affects short-term retention. The results are consistent with several other studies of MTL lesion cases using different types of VSTM tasks which report impairments specifically in binding object identity to location (Hannula et al., 2006, Libby et al., 2014, Olson et al., 2006, Watson et al., 2013). In addition, recent evidence points to a role for the hippocampus also in unconscious relational binding (Duss et al., 2014). These considerations suggest that the distinction between long- and short-term, conscious and unconscious memory systems may be less clear than traditionally considered (Cohen and Eichenbaum, 1993, Hannula et al., 2006, Olson et al., 2006, Ranganath and Blumenfeld, 2005).

It is now established that paired-associate learning (PAL) in sporadic AD is sensitive to disease progression (Fowler, Saling, Conway, Semple, & Louis, 2002). Activation of the human hippocampus has been demonstrated during the encoding phase of CANTAB version of the PAL which involves associating an object identity with a location (De Rover et al., 2011). People with mild cognitive impairment (MCI) have decreased hippocampal activation with increasing memory load, whereas by contrast healthy controls show the opposite pattern (De Rover et al., 2011). Furthermore, impaired performance on the CANTAB PAL correlates with hippocampal volume loss in MCI (Kéri, Szamosi, Benedek, & Kelemen, 2012). A recent neuroimaging study has also shown that MTL structures play an important role in associating different stimuli (in this case objects and scenes) when retrieving them from memory (Staresina, Cooper, & Henson, 2013). Together, these findings suggest that binding information in memory might be an important function of MTL regions which seem to make an important contribution to performance on PAL tasks. A common deficit in binding information might therefore underpin performance on PAL as well as the delayed reproduction task presented here.

The current study has several limitations. First, it might be argued that the VSTM deficits in mutation carriers might be confounded by perceptual difficulties. This is more plausible for the *symptomatic* FAD cases, who showed deficits in memory for object identity, but seems less likely to influence the results from the *asymptomatic* gene carriers because their identification performance was unimpaired and binding deficits were mainly observed for long delays (perceptual impairment should affect both delays). Second, the sample size was relatively small due to the rarity of FAD and the limited number of symptomatic individuals who were able to perform the task to a reasonable level. As a result, the mutation carriers in our study were pooled from pedigrees with different *PSEN1* and *APP* mutations. Therefore, it is not possible to draw conclusions about individual genotypes or to assess differences between *PSEN1* and *APP* mutations. However, given that our findings were achieved with a heterogeneous genetic cohort, it is likely that the effect is related to hippocampal dysfunction, common to all FAD mutations, rather than some gene-specific property. Third, a recent study (Pertzov et al., 2015) found that normal ageing is also associated with increased swap error rates– potentially raising concerns over the specificity of the impairment we found. However, a more detailed analysis of swap errors-one that corrects for errors that could be attributed to participants not remembering the identity of the fractals-shows no age-related impairment. Direct comparison of the FAD and healthy ageing data suggests that swap error rates-both corrected and uncorrected-are higher in the FAD cohort (For more details see Supplementary Materials).

In summary, we have shown that failure in object-location binding in VSTM is an early cognitive feature of FAD, observable before impairment in object identification, localization and standard neuropsychology measures of WM and long-term memory appear. Consistent with the concept that the hippocampus is fundamentally engaged in relational binding in memory, we found that hippocampal volume significantly predicted the degree of binding errors in mutation carriers. Abnormal object-location binding might therefore be a sensitive cognitive biomarker for early MTL pathology including AD.

## Figures and Tables

**Table 1:** Characteristics of FAD gene carriers and age-matched controls. Mean values are given with SDs.

Group	Age (yrs)	Males (%)	Education (yrs)	MMSE (/30)	Anxiety HAD scale (/21)	Depression HAD scale (/21)	NART (/50)	Years to parental age of symptom onset
Controls (N = 50)	36.9 (4.1)	50%	15.7 (2.6)	29.5 (.9)	6.1(3.8)	3.1 (2.8)	31 (9.0)	NA
Asymptomatic carriers (N = 12)	37.2 (4.4)	25%	13.4 (2.4)	29.4 (.9)	5 (4.2)	1.3 (2.2)	28.3 (9.3)	8.5 (3.8)
*p* value	.85	.75	.01	.74	.43	.02	.37	NA

Group	Age (yrs)	Males (%)	Education (yrs)	MMSE (/30)	Anxiety HAD scale (/21)	Depression HAD scale (/21)	NART (/50)
Controls (N = 28)	46.8 (6.9)	46%	14.3 (2.6)	29.7 (.5)	4.9 (3.5)	2.6 (2.7)	31.9 (10.3)
Symptomatic carriers (N = 8)	47.4 (10.2)	63%	13.9 (3.1)	25.8 (3.4)	6.3 (4.4)	2.4 (2.3)	24.3 (12.6)
*p* value	.89	.69	.74	<.001	.47	.84	<.001

**Table 2:** Neuropsychology results of FAD gene carriers and age-matched controls. Mean values are given with SDs.

Test	Controls (*N* = 50)	Asymptomatic carriers (*N* = 12)	*p* Value or C.I. estimates by boot strapping
IQ (WASI)	116.9 (11.9)	103.6 (13.2)	<.001
RMT Words/50	48.4 (2.2)	47 (2.6)	−3.1 to .2
RMT Faces/50	41.6 (4.9)	43.3 (3.4)	.21
WMS-LM immediate/25	16.4 (4.2)	14.3 (3.8)	.10
WMS-LM delayed/25	14.9 (3.9)	13.5 (3.3)	.25
Rey (delay:copy)	.69 (.1)	.61 (.2)	.11
Digit span forward max/8	7.2 (1.1)	6.9 (1.0)	−.34 to .09
Digit span backward max/7	5.31 (1.2))	5.42 (1.0)	.36
Spatial span forward max/9	5.9 (1.0)	5.3 (1.2)	−1.5 to .04
Spatial span backward max/9	5.5 (1.0)	5.4 (1.2)	.96
Letter fluency (FAS)	46.7 (11.0)	43.8 (5.8)	.57
Stroop	28.1 (10.4)	32.8 (10.2)	.22
Trail making	30.7 (20.5)	34.4 (14.8)	−8.6 to 13.2
Category fluency	39.4 (8.3)	38.4 (11.7)	.94
GNT/30	20.7 (4.7)	19.6 (4.5)	.85
GDA/24	16.2 (5.3)	15.6 (4.3)	.9
VOSP (object decision)/20	17.7 (1.7)	18.4 (1.3)	.13
Digit symbol	39.4 (8.3)	38.4 (11.7)	.18

Test	Controls (*N* = 28)	Symptomatic carriers (*N* = 8)	*p* Value or C.I. estimates by boot strapping
IQ (WASI)	116.7 (11.1)	89.4 (21.6)	<.001
RMT words/50	48 (1.9)	38 (8.5)	−10.4 to −7.1
RMT faces/50	42.6 (4.2)	40.3 (3.7)	.40
WMS-LM immediate/25	15.1 (3.8)	8.4 (3.7)	.01
WMS-LM delayed/25	14.5 (3.5)	5.5 (3.6)	<.001
Rey (delay:copy)	.67 (.14)	.36 (.22)	<.001
Digit span forward max/8	7.0 (1.1)	5.9 (1.6)	.15
Digit span backward max/7	5.1 (1.2)	4.3 (1.6)	.005
Spatial span forward max^a^/9	5.5 (.7)	4.3 (1.1)	.003
Spatial span backward max/9	5.1 (.8)	4.7 (1.1)	.19
Letter fluency (FAS)	46.4 (11.2)	35.1 (11.1)	.051
Stroop	28.7 (11.6)	56.9 (43.3)	.02
Trail making	35.2 (23.6)	89.1 (75.8)	13.9 to 95.2
Category fluency	39.3 (8.3)	31 (7.3)	.053
GNT/30	22.4 (4.7)	18.4 (7.2)	.35
GDA/24	16.4 (4.5)	9.5 (5.9)	.003
VOSP (object decision)/20	18.1 (2.0)	17.8 (1.7)	.86
Digit symbol	56.6 (8.9)	38.6 (14.5)	.001

RMT: recognition memory test.

WMS-LM: Wechsler Memory Scale-logical memory.

GNT: Graded naming test.

GDA: Graded difficulty arithmetic test.

VOSP: Visual Object and Spatial Perception.

a Scores from spatial span forward maximum underwent cube transformation.
