# Supplementary material for: Visual short-term memory binding deficit in familial Alzheimer's disease
Source: Cortex. 2016 May;78:150–64. doi: 10.1016/j.cortex.2016.01.015 (PMC4865502; doi:10.1016/j.cortex.2016.01.015)
Supplement: Supplementary file 1 [file mmc1.doc]

**SUPPLEMENTARY MATERIAL**

**Visual short-term memory binding deficit in familial Alzheimer’s disease**

**Y Liang et al**

**Supplementary Methods**

**Selection of control groups**

To select subsets of controls who were age-matched to the asymptomatic and symptomatic mutation carriers groups respectively, we used box plots (showing the median, interquartile range and total range for age) to guide us in terms of the appropriate age cut-offs to apply. Guided by the box plots, controls older than 47 years of age were excluded when selecting individuals to be age-matched to the asymptomatic group and controls younger than 39 years of age were excluded when selecting those to be age-matched to the symptomatic group. We then performed t-tests to check that the resultant two subsets of controls were well matched to the two gene carrier groups in terms of the group means and standard deviations. The selection of the controls was solely based on age criteria and no reference to task performance.

**Neuropsychology tests**

Apart from the standard tests of working memory and long-term memory described in the main text, the following were also included in the neuropsychology battery: verbal fluency (sum total of words generated in one minute beginning with letters F, A and S respectively) (Spreen and Strauss, 1998), Stroop (difference in time taken to complete the conflict and word conditions) (Stroop, 1935), Trail Making Test (difference in time taken to complete TMB and TMA) (Reitan, 1958), category fluency (sum total of animal and vegetable names generated in one minute respectively) (Spreen & Strauss, 1998), Graded Naming Test (GNT) (McKenna and Warrington, 1983); Graded Difficulty Arithmetic Test (GDA) (Jackson & Warrington, 1986); object decision test from the Visual Object and Space Perception battery (VOSP) (Warrington and James, 1991) and digit symbol test (Wechsler, 1981).

**Visual short-term memory (VSTM) experiment**

**Visual stimuli**

**
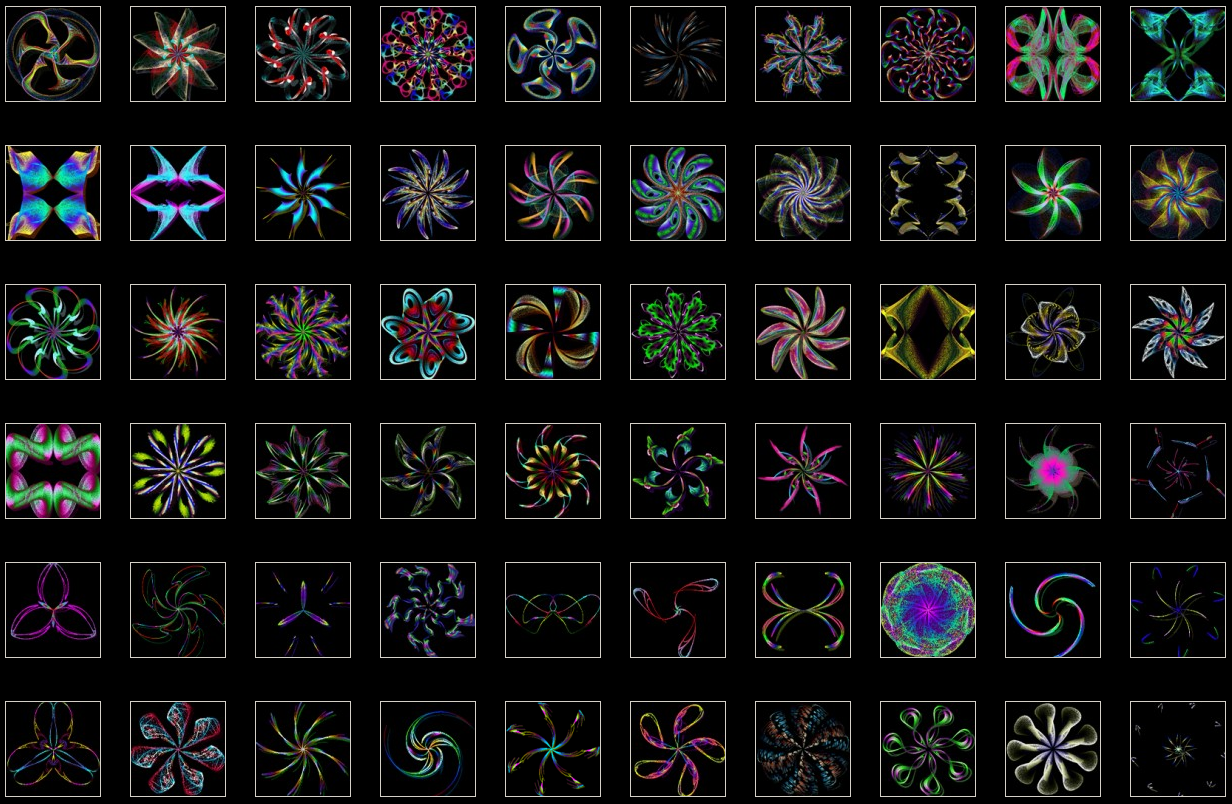
**

**Supplementary Figure 1. Visual stimuli used.**

60 colored fractals on black background were used. Symmetrical fractals were generated using code provided in Sprott's Fractal Gallery (<http://sprott.physics.wisc.edu/fractals.htm>). Fractals were resized to have maximum width and height of 120 pixels (~4° of visual angle in experimental set-up).

**Calculation of swap error corrected for chance**

In order to ensure that the increased number of swap errors did not simply result from increased gross localisation errors, i.e. objects localised further away from their original location might generate more (apparent) swap errors simply by chance, we performed the following calculation. For each trial, we calculated the probability of obtaining swap errors by chance by computing all potential locations with the same absolute distance of error from the original target location at all possible angular deviations (using steps of 1^0^) with the proviso that a simulated location had to be within the screen dimensions and the invisible margins used for generating the display. The chance probability of obtaining a swap error is therefore the number of simulated locations within our 4.5^0^ threshold perimeter around non-targets, divided by all possible valid, simulated locations (Pertzov et al., 2012, 2013). We performed this calculation for every trial using its specific distance of error from the target item. Then, we subtracted the number of swap errors predicted by chance from the measured number of swap errors. This gave us a measure of **swap errors corrected for chance.**

**Statistical analysis for VSTM outcomes**

Analysis of identification performance used a logistic regression model for the odds of choosing the correct object in the test array. Analysis of localization performance used a linear regression model for natural logarithm of gross localization error and localization precision (using “nearest neighbour control” analysis). Analysis of swap errors used a linear regression for square root of proportion of swaps and square root of proportion of swaps controlled for chance. A square root transformation was necessary because of the skew in the proportion of swap errors. The same transformation was used for both swap analyses to allow comparison of performance with and without control for chance.

For each outcome, performance was compared between the entire FAD group and all controls, asymptomatic individuals and aged-matched controls and symptomatic individuals and age-matched controls. For each outcome, we first tested for main effects of group, delay and block. For object identity and gross mislocalization error, we also tested for main effect of item number. Further analysis then explored two-way interactions between group and each condition, namely delay, block and number of items (in the case of object identity and gross mislocalization error). This allowed us to examine whether any between-group differences in performance depended on the task condition. Where a significant two-way interaction was found or where there was an *a priori* hypothesis, we also examined three-way interactions between group, delay and block or item number. Results of interactions are only presented if they were statistically significant or relevant to the overall interpretation of the experiment. All analyses of the outcomes of VSTM experiments were adjusted for the effects of NART and sex. The analyses corrected for NART- a marker of education - but not IQ as NART has previously been shown to be relatively resistant to the effect of AD (Law and O’Carroll, 1998) whereas, importantly, IQ is not (Fox et al, 1998; Godbolt et al 2004, 2005). Were we to correct for IQ, any effect related to AD itself could be falsely diminished. Furthermore, for both the FAD mutation carriers as a whole and for controls, there is actually a statistically significant association between total IQ and NART (see Supplementary Results below). Robust standard errors were used to allow for repeated measures within the same individual.

**Statistical analysis for associations between hippocampal volumes and neuropsychology tests**

The associations between neuropsychology tests for memory and working memory and hippocampal volume were evaluated using linear regression with inclusion of group, hippocampal volume and its interaction with group as predictors. Where necessary, bootstrap confidence intervals were provided for the association between hippocampal volume and neuropsychology tests. All analyses adjusted for age, sex and total intracranial volume.

**Supplementary Results**

**Baseline characteristics**

The FAD cohort as a whole had on average fewer years of formal education and lower MMSE, depression and NART scores.

**Supplementary Table 1**. **Baseline characteristics of all FAD cases and controls**

Mean values are given with SDs.

| **Group** | **Age**  **(yrs)** | **Males** | **Education**  **(yrs)** | **MMSE**  **(/30)** | **Anxiety**  **HAD scale**  **(/21)** | **Depression**  **HAD scale**  **(/21)** | **NART**  **(/50)** |
| --- | --- | --- | --- | --- | --- | --- | --- |
| **Controls (N=62)** | 40.1 (7.9) | 31 | 15.5 (2.7) | 29.5 (0.8) | 5.9 (3.8) | 3.13 (2.88) | 32.3 (9.1) |
| **FAD**  **(N=20)** | 41.3 (8.7) | 10 | 13.6 (2.6) | 28.0 (2.8) | 5.5 (4.2) | 1.68 (2.24) | 26.7 (10.6) |
| **P value** | 0.61 | 1 | 0.01 | 0.01 | 0.68 | 0.03 | 0.04 |

**Neuropsychology results**

**All FAD cases**

On average, FAD participants were significantly worse than controls at current IQ, RMT Words, WMS-logical memory immediate and delayed conditions, Rey complex figure, spatial span forward maximum, Stroop, Trail making, Graded Difficulty Arithmetic test and Digit symbol test (Supplementary Table 2).

**Supplementary Table 2 Neuropsychology results of all FAD cases and controls**

Mean values are given with SDs.

| **Test** | **Controls**  **(N=62)** | **FAD**  **(N=20)** | **P value or C.I. estimates by boot strapping** |
| --- | --- | --- | --- |
| **IQ (WASI)** | 117.4 (11.8) | 97.9 (18.0) | <0.001 |
| **RMT Words /50** | 48.4 (2.1) | 43.4 (7.4) | -7.7 to -1.88 |
| **RMT Faces /50** | 42.2 (4.8) | 42.1 (3.7) | 0.58 |
| **WMS-LM immediate /25** | 16.4 (4.02) | 11.9 (4.73) | 0.001 |
| **WMS-LM delayed^*^ /25** | 15.0 (3.83) | 10.3 (5.28) | 0.001 |
| **Rey^*^ (delay:copy)** | 0.69 (0.12) | 0.52 (0.24) | 0.002 |
| **Digit span forward max /8** | 7.20 (1.1) | 6.5 (1.4) | -0.95 to 0.19 |
| **Digit span backward max/7** | 5.26 (1.15) | 4.95 (1.39) | 0.81 |
| **Spatial span forward max /9** | 5.81 (0.97) | 4.95 (1.27) | -1.49 to -0.34 |
| **Spatial span backward max /9** | 5.42 (1.0) | 5.16 (1.17) | 0.49 |
| **Letter fluency (FAS)** | 46.5 (10.4) | 40.3 (9.1) | 0.13 |
| **Stroop** | 27.7 (10.7) | 42.5 (29.3) | 7.60 to 14.0 |
| **Trail making** | 31.3 (19.4) | 56.3 (54.8) | 5.05 to 47.2 |
| **Category fluency** | 40.0 (8.13) | 35.5 (10.6) | 0.20 |
| **GNT /30** | 21.4 (4.78) | 19.1 (5.58) | 0.81 |
| **GDA /24** | 0.69 (0.12) | 0.52 (0.24) | 0.002 |
| **VOSP (object decision) /20** | 17.9 (1.76) | 18.2 (1.46) | 0.28 |
| **Digit symbol** | 62.2 (11.1) | 50.6 (18.3) | 0.003 |

**Legend:**

RMT: recognition memory test

WMS-LM: Wechsler Memory Scale-logical memory

GNT: Graded naming test

GDA: Graded difficulty arithmetic test

VOSP: Visual Object and Spatial Perception

*Scores from WMS-LM delayed and Rey complex figure underwent square transformation

**Relationship between NART and IQ (WASI)**

There is a statistically significant association between total IQ and NART in both the FAD cohort (0.49 point increase in NART score for every point increase in total IQ, p<0.001) and in controls (0.54 point increase in NART for every point increase in total IQ, p<0.001). But there is no significant interaction in the relationship between NART and IQ between the two groups (0.05 point difference in NART for every 1 point difference in IQ, p=0.6)).

**Associations between standard neuropsychology tests and hippocampal volumes**

We examined associations between hippocampal volume and the following tests of memory and working memory: RMT words, RMT faces, WMS-LM immediate and delayed conditions, Rey complex figure (delayed:copy), digit span and spatial span (forward and backward).

For the entire FAD group, hippocampal volume significantly predicted performance in RMT words (coefficient = 5.78, p<0.05), WMS-LM delayed (coefficient = 2.91, p = 0.04) and Rey complex figure (coefficient = 0.19, p = 0.001) with significant interactions between controls and FAD group for RMT words (coefficient = 4.29, p < 0.05) and Rey complex figure (coefficient = 0.15, P = 0.02) but not for WMS-LM delayed (coefficient = 2.90, p = 0.07). None of the other tests of memory and working memory showed significant associations with hippocampal volumes in the entire FAD case.

For RMT words and WMS story-delayed version, when the asymptomatic and symptomatic gene carrier groups were examined separately, there were no significant associations between the test scores and hippocampal volumes in either the former (RMT words: coefficient = -0.04, p > 0.05; WMS story-delayed coefficient = -0.97, P = 0.58) or latter (RMT words: coefficient = 10.3, p > 0.05; WMS story-delayed coefficient = 2.13, P = 0.45). It is possible that the association between RMT words score and hippocampal volume found in the entire FAD group could be driven by differences in hippocampal volumes between the two gene carrier groups. However, there was a significant correlation between Rey complex figure and hippocampal volume in the asymptomatic gene carriers (coefficient = 0.18, P = 0.006) but not in the symptomatic individuals (coefficient = -0.16, P = 0.27) or controls (coefficient=0.03, p=0.39) with significant interactions between controls and asymptomatic gene carriers (coefficient = 0.15, p = 0.02) and between asymptomatic and symptomatic gene carriers (coefficient = -0.35, p = 0.03). This suggests that, for Rey complex figure, the correlation with hippocampal volume found in the entire FAD cases was likely to be driven by asymptomatic gene carriers.

**Variation in performance across fractals**

In our stimuli set, the visual saliency of the fractals varies from stimulus to stimulus. We have examined how this variation affects subjects performance by grouping the data according to each probed fractal (shown on the x-axis of the plots below) and investigated if subjects’ performance differed consistently in the three main measures: identification, localization and swap error.


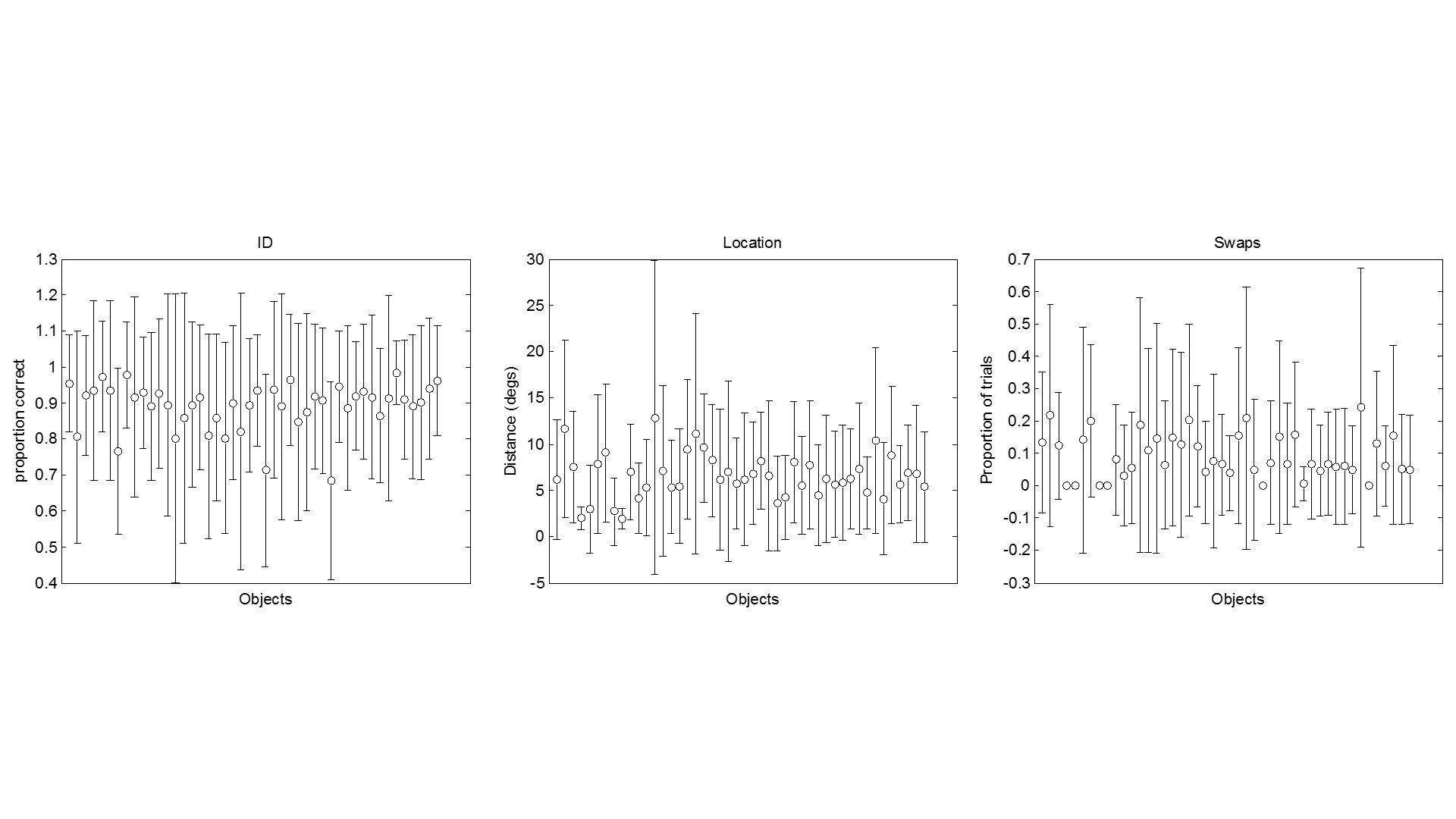


**Supplementary Figure 2. Variation across stimuli.**

Identification, localization and swaps performance for the different probed fractals (x –axis). Averaged values across participants (all FAD and normal) are in circles, error bars represent standard deviations across participants.

ANOVA treating the participants as repeated measures shows clear variation across objects (Fs > 5, Ps < 0.001). Note that a few fractals were never swapped by any participant. Thus, there are consistent differences in reporting the fractals, probably due to differences in visual complexity and saliency.

However, generating a database of stimuli that are equal in their physical complexity is challenging as practically ALL possible features would have to be comparable (e.g. color distinctiveness, convexity etc). More importantly, for the claim made in the manuscript, all participants (FAD and healthy) were presented with the same set of stimuli (in random order of trials) so any difference between the groups – as we report – cannot be attributed to variation in visual saliency. Future designs should study the advantage of using a more homogeneous set of stimuli.

**Specificity of our results: aging, FAD and swap errors**

We have recently published age-related, cross-sectional performance data using the same change detection task (Pertzov et al., 2015). Below is a figure plotting the regression line associating age and swap errors for the long delay condition in the first block of the task. Note in the original study (Pertzov et al., 2015), the swap error rates were collapsed across blocks and delays. For comparison we plotted the averaged swap error rates of the FAD mutation carriers from the current study. Based on visual assessment, for both corrected (A) and total (uncorrected) swap error rates (B), there was at least a 67% chance that the averaged mean for the FAD group differed from that for the healthy aging population. The correction takes into account the fact that some swap errors would have occurred as participants chose the correct target object by chance. For a detailed explanation of how the correction was performed, please see previous manuscript (Pertzov 2015).


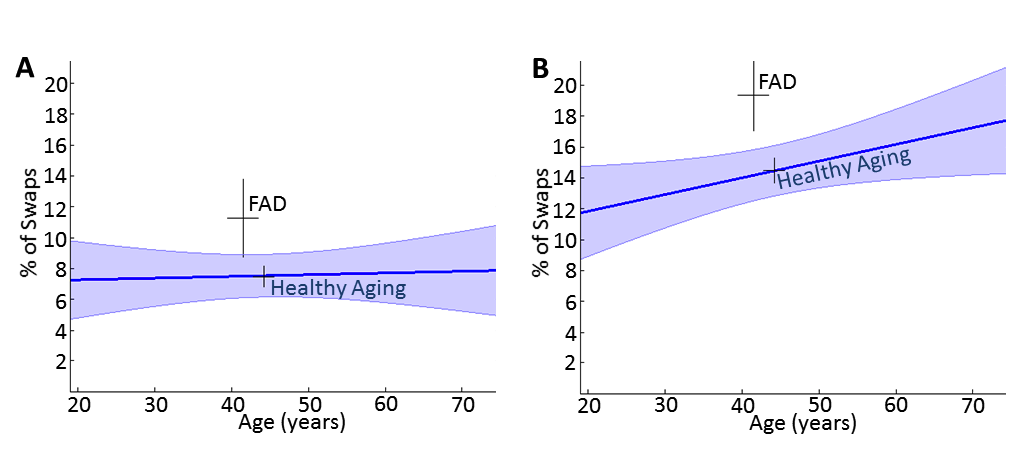


**Supplementary Figure 3. Swap error rates in FAD and healthy aging.**

1. Linear regression (blue) and regression confidence intervals (shaded blue) between age and overall proportion of trials where swap errors *corrected for identification errors* occurred. (B) Linear regression (blue) and regression confidence intervals (shaded blue) between age and overall proportion of trials where uncorrected swap errors occurred. Grey vertical and horizontal lines represent the standard errors of the mean averaged swap errors and mean age respectively for FAD and healthy aging. All swap errors were from the longer delay condition in the first block of the delayed reproduction task.

**References**

Fox, N. C., Warrington, E. K., Seiffer, A. L., Agnew, S. K., & Rossor, M. N. (1998).

Presymptomatic cognitive deficits in individuals at risk of familial Alzheimer's disease: a longitudinal prospective study. Brain, 121, 1631e1639.

Godbolt, A. K., Cipolotti, L., Anderson, V. M., Archer, H., Janssen, J. C., Price, S., et al. (2005). A decade of pre-diagnostic assessment in a case of familial Alzheimer's disease: tracking progression from asymptomatic to MCI and dementia. Neurocase, 11, 56e64.

Godbolt, A. K., Cipolotti, L., Watt, H., Fox, N. C., Janssen, J. C., & Rossor, M. N. (2004). The natural history of Alzheimer disease. Archives of Neurology, 61, 1743e1748.

Jackson, M., & Warrington, E. K. (1986). Arithmetic skills in patients with unilateral cerebral lesions. Cortex, 22, 611e620.

McKenna, P., & Warrington, E. (1983). The Graded Naming Test. Windsor: NFER-Nelson.

Reitan, R. (1958). Validity of the trail making test as an indicator of organic brain damage. Perceptual and Motor Skills, 8, 271e276.

Spreen, O., & Strauss, E. A. (1998). Compendium of neuropsychological tests (2nd ed.). New York, NY: Oxford University Press.

Stroop, J. R. (1935). Studies of interference in serial verbal reactions. Journal of Experimental Psychology, 18, 643e662.

Warrington, E. K., & James, M. (1991). The visual object and space perception battery. Bury St Edmunds (UK): Thames Valley Test Company.

Wechsler, D. (1981). Manual for Wechsler adult intelligence scale- revised. New York: Psychological Corporation.
